# A Comprehensive Review of Food and Drug Administration (FDA)-Cleared Virtual Reality Technologies in Radiology

**DOI:** 10.7759/cureus.85440

**Published:** 2025-06-05

**Authors:** Amy Avakian

**Affiliations:** 1 Diagnostic Radiology, Ross University School of Medicine, Bridgetown, BRB

**Keywords:** ai, ai in radiology, diagnostic radiology, radiology, virtual reality, virtual reality in radiology, vr, vr diagnostic imaging, vr in healthcare, vr in radiology

## Abstract

Virtual reality (VR) is emerging as a clinically relevant tool in diagnostic radiology, offering immersive environments for image interpretation, medical education, and preoperative planning. Despite the field’s continued reliance on two-dimensional (2D) monitors to assess inherently three-dimensional (3D) imaging data, immersive technologies remain underutilized in clinical workflows. As of 2024, the U.S. Food and Drug Administration (FDA) has cleared 69 devices categorized under virtual and augmented reality. This review focuses on the seven VR systems with direct applications in radiology: Ceevra Reveal 3+ (Ceevra, Inc., San Francisco, CA, USA), VSI HoloMedicine (apoQlar medical GmbH, Hamburg, Germany), Dextroscope (Volume Interactions Pte Ltd, Singapore), Dextrobeam (Volume Interactions), V3D-Colon (Viatronix, Inc., Stony Brook, NY, USA), V3D-Explorer (Viatronix), and V3D-Vascular (Viatronix). These platforms enable users to visualize, manipulate, and interpret radiological data in fully immersive 3D space, enhancing diagnostic precision, procedural planning, and interdisciplinary collaboration. Drawing on regulatory documentation and peer-reviewed evaluations, this article assesses the clinical functions, implementation challenges, and future potential of these technologies. As immersive tools gain regulatory momentum and technical refinement, VR is positioned to expand radiology’s diagnostic capabilities and reshape its educational and clinical landscape.

## Introduction and background

Radiology has consistently been a leader in integrating emerging technologies into clinical care. From the introduction of computed tomography (CT) and magnetic resonance imaging (MRI) to the widespread adoption of picture archiving and communication systems (PACS), the specialty has evolved in step with innovation. Yet despite interpreting inherently three-dimensional (3D) data, radiologists continue to rely on two-dimensional (2D) screens for image review. This spatial limitation requires cognitive reconstruction of anatomy, a process that can contribute to diagnostic inefficiencies and errors [1].

Virtual reality (VR) offers a transformative solution. By enabling immersive visualization of imaging data, VR allows users to interact with volumetric datasets in real-time 3D space. In such environments, radiologists can rotate, segment, and explore anatomical structures more intuitively than in conventional PACS workstations. Early applications of VR in medicine have demonstrated value in surgical simulation, patient-specific planning, and anatomy education [2,3]. However, its adoption in diagnostic radiology has been slower, partly due to concerns over image resolution, workflow disruption, and regulatory approval.

This landscape is changing. As of 2024, the U.S. Food and Drug Administration (FDA) has cleared 69 devices categorized under virtual and augmented reality. While the FDA has cleared over 60 devices under the broader umbrella of virtual and augmented reality technologies, this review focuses exclusively on the seven FDA-cleared VR platforms with direct, standalone diagnostic or preoperative visualization functionalities explicitly designed to process and render radiologic imaging data (e.g., CT, MRI, positron emission tomography (PET)). These systems are fully virtual and have applications specifically in radiology: Ceevra Reveal 3+ (Ceevra, Inc., San Francisco, CA, USA), VSI HoloMedicine (apoQlar medical GmbH, Hamburg, Germany), Dextroscope (Volume Interactions Pte Ltd, Singapore), Dextrobeam (Volume Interactions), V3D-Colon (Viatronix, Inc., Stony Brook, NY, USA), V3D-Explorer (Viatronix), and V3D-Vascular (Viatronix) [1]. These platforms enable users to visualize, manipulate, and interpret radiological data in fully immersive 3D space, enhancing diagnostic precision, procedural planning, and interdisciplinary collaboration. These systems vary in design and functionality but share a common goal: to enhance the way imaging data is visualized, interpreted, and applied in clinical decision-making.

While other FDA-cleared AR/VR tools may interface with imaging data in broader surgical or therapeutic contexts, they often do so as ancillary features or lack core functionalities for immersive image interpretation or 3D anatomical navigation. To ensure clinical relevance and analytical depth, this review limits its scope to the devices whose primary function aligns directly with radiologic visualization, education, and diagnostic planning

These advancements are exemplified by a growing number of FDA-cleared VR platforms tailored for radiologic use. Table 1 summarizes seven currently approved devices, outlining their clinical indications and technical specifications [1]. This overview illustrates the expanding role of immersive technologies in diagnostic imaging, procedural planning, and medical education.

**Table 1:** Current FDA-cleared virtual reality (VR) devices for use in radiology Source: U.S. Food and Drug Administration [1]

Device Name	Manufacturer	FDA Clearance Date	Primary Use in Radiology
Ceevra Reveal 3+	Ceevra, Inc.	2023	3D visualization of radiological data for surgical planning and patient communication
VSI HoloMedicine	apoQlar medical GmbH	2022	Immersive 3D visualization of medical images for preoperative planning
Dextroscope	Volume Interactions Pte Ltd	2002	VR environment for neurosurgical planning and radiology education
Dextrobeam	Volume Interactions Pte Ltd	2002	Collaborative VR console for 3D medical imaging analysis and education
V3D-Colon	Viatronix, Inc.	2002	Virtual colonoscopy software for 3D visualization of CT colonography data
V3D-Explorer	Viatronix, Inc.	2002	General 3D visualization software for CT, MR, and PET images
V3D-Vascular	Viatronix, Inc.	2002	3D visualization and quantification of vascular structures from imaging data

## Review

Clinical applications of FDA-cleared VR in radiology

Seven VR platforms currently cleared by the FDA for clinical use in radiology offer capabilities across three major domains: immersive image interpretation, procedural planning, and radiologic education. These devices integrate immersive visualization into various radiology workflows, supporting more intuitive understanding of complex anatomy, enhanced interprofessional collaboration, and improved teaching of imaging principles.

Immersive Image Interpretation

Immersive VR platforms enhance the way radiologists interact with complex datasets, particularly for volumetric modalities like CT, MR, and PET. V3D-Explorer, developed by Viatronix, enables stereoscopic rendering of multi-modality data, allowing users to manipulate images in real time with tools for measurement, segmentation, and cross-sectional review [2]. Its intuitive interface makes it well-suited for both clinical and educational applications.

V3D-Vascular, another Viatronix product, focuses specifically on vascular imaging. It reconstructs detailed vessel geometries from CT angiograms and enables users to assess stenoses, bifurcations, and tortuosity with increased depth perception and clarity [3]. This spatial advantage has clinical implications. In a pilot study, Kim et al. found that VR-based planning with V3D-Vascular improved diagnostic confidence and preoperative preparation for vascular interventions compared to conventional 2D workflows [4].

Procedural and Preoperative Planning

Surgical planning often requires radiologists and surgeons to interpret complex anatomy collaboratively. VR platforms such as VSI HoloMedicine and Ceevra Reveal 3+ enable that interaction by transforming imaging data into manipulable 3D holographic or stereoscopic models.

VSI HoloMedicine, developed by apoQlar medical GmbH, allows holographic projection of segmented CT and MRI data in shared 3D space [5]. It supports intuitive hand gestures for manipulating anatomy and integrates annotations and segmentation tools for surgical rehearsal. In a feasibility study, Sun et al. found that its use led to a 30% reduction in preoperative planning time, with surgeons reporting improved comprehension of spatial relationships [6].

Ceevra Reveal 3+ similarly transforms Digital Imaging and Communications in Medicine (DICOM) data into high-resolution anatomical models that can be explored collaboratively in a VR environment [7]. The platform has been adopted in surgical oncology and reconstructive planning workflows. Smith reported improved interdepartmental communication and more precise localization of tumor margins when the platform was incorporated into case reviews [8].

Neurosurgical Visualization and Simulation

Dextroscope represents one of the earliest VR platforms designed for clinical imaging. It enables users to virtually navigate through neuroanatomy, perform simulated craniotomies, and rehearse complex skull base or spine procedures. Though not widely used today, its role as a forerunner in immersive surgical imaging remains notable. Kockro et al. described Dextroscope’s long-term impact on neurosurgical education and planning, particularly in tumor resection and vascular access strategies [9].

Radiologic Education and Virtual Training

Beyond diagnosis and planning, FDA-cleared VR platforms have shown strong value in teaching radiologic anatomy and interpretation skills. V3D-Colon offers a fully immersive virtual colonoscopy experience using real CT colonography datasets [10]. This tool enables endoluminal navigation, lesion detection practice, and anatomical orientation training. In a diagnostic study, Pickhardt reported that trainees using V3D-Colon had improved polyp detection and retention of spatial anatomy compared to traditional axial review [11].

V3D-Explorer also supports radiology education by offering real-time manipulation of datasets in 3D. In a teaching hospital study, Abuzaid et al. found that trainees using the platform performed significantly better on anatomy assessments and showed greater engagement during case review sessions [12].

VSI HoloMedicine further contributes to immersive learning by providing volumetric overlays of anatomy and pathology. A recent study by Tene et al. found that students exposed to VR-based radiology education demonstrated improved test performance and greater confidence interpreting cross-sectional images [13].

Comparative literature discussion

Although the clinical use of immersive VR in radiology is expanding, large-scale comparative studies remain limited. Nonetheless, early evaluations suggest that FDA-cleared platforms may improve spatial understanding, interprofessional communication, and learning outcomes when compared to conventional 2D imaging workflows.

Usability and Ergonomics

One consistent finding in the literature is the ease of adoption of modern VR platforms. Tools such as V3D-Explorer and VSI HoloMedicine have been described as intuitive, with minimal training required for effective use [2,5]. However, ergonomic concerns remain. In a study by Baniasadi et al., radiologists reported eye strain, neck discomfort, and reduced attention span after extended use of head-mounted VR displays, particularly during sessions exceeding 30 minutes [14]. These issues highlight the need for ergonomic optimization and session time guidelines in clinical workflows.

Diagnostic Performance

Comparative studies evaluating diagnostic accuracy between VR and 2D PACS systems suggest that immersive environments may enhance reader performance. In a prospective study, Shields et al. found that radiologists interpreting vascular abnormalities with V3D-Vascular had significantly greater interobserver agreement and diagnostic confidence compared to those using traditional 2D reconstructions [15].

Similarly, Benchoufi et al. evaluated radiologists’ performance in complex musculoskeletal and neurologic cases using immersive VR tools. They found that intra- and interobserver consistency improved when readers used 3D platforms, especially for spatially complex findings such as displaced fractures or midline shift [16]. These improvements suggest that immersive visualization may reduce diagnostic ambiguity, particularly in anatomically dense regions.

Cost-Effectiveness and Implementation Feasibility

Financial considerations remain a key barrier to VR adoption in healthcare. While hardware costs are declining, integration with existing systems and IT support still require significant institutional commitment. In a health technology assessment, Moro et al. modeled the cost-effectiveness of immersive surgical planning technologies, concluding that VR platforms were economically viable in high-complexity procedural environments, particularly in oncology and neurosurgery [17]. Their model emphasized savings from reduced intraoperative time, fewer surgical complications, and improved patient education.

Communication and Patient Engagement

Immersive technologies also offer potential benefits in enhancing patient understanding. In a study by Jiang et al., patients who received VR-guided surgical previews via Ceevra Reveal 3+ demonstrated better comprehension of their condition and treatment plan, leading to more informed consent and reduced decisional conflict [18]. This application may have broader implications in shared decision-making, especially in high-stakes or anatomically complex procedures.

Similarly, Lyuksemburg et al. showed that cross-specialty case reviews conducted in VR environments fostered clearer communication between radiologists, surgeons, and trainees. In oncology cases involving hepatic and pelvic tumors, collaborative planning using VR platforms resulted in more unified surgical strategies and fewer intraoperative revisions [19].

Future directions and integration challenges

As virtual reality tools become more accessible and clinically capable, the next phase of adoption in radiology will depend on several converging developments: stronger validation studies, seamless technical integration, and cultural acceptance within clinical practice.

Validation Through Rigorous Study Design

While early data suggest diagnostic, educational, and procedural advantages of immersive imaging, most studies to date have been limited in scope, sample size, or generalizability. Future research must include randomized controlled trials evaluating time to diagnosis, interpretation accuracy, intra- and interobserver variability, and downstream patient outcomes. Additionally, multicenter trials comparing immersive and conventional workflows in diverse radiologic subspecialties will be necessary to clarify real-world value.

Standardizing evaluation metrics will also be critical. Current studies vary widely in how they assess outcomes, ranging from subjective feedback to performance-based metrics. A unified framework could help facilitate device comparisons and accelerate evidence-based adoption.

Integration with PACS, Artificial Intelligence (AI), and Clinical Workflow

For VR tools to be incorporated meaningfully into diagnostic practice, they must be interoperable with existing infrastructure. Current limitations in PACS integration often require separate workstations or middleware to render DICOM data in VR, adding friction to clinical use. Developers must prioritize Health Level 7 (HL7) and DICOMweb compatibility to streamline access.

Further synergy with AI may unlock even greater utility. For example, AI-assisted segmentation within VR platforms could allow users to interact directly with labeled tumors, vessels, or functional regions. Such integration would not only accelerate interpretation but also support personalized medicine through radiomic analysis and predictive modeling.

Cultural and Institutional Adoption

Beyond technical readiness, the cultural integration of VR into radiology will require educational reform and institutional buy-in. Most radiologists are not trained to interpret in immersive environments. Introducing VR modules into medical school anatomy labs, radiology clerkships, and residency curricula may help normalize the technology early and reduce resistance to adoption.

From an administrative perspective, demonstrating return on investment will be essential. This includes not only cost savings, as shown by Moro et al. [17], but also improvements in patient outcomes, clinician efficiency, and satisfaction. Institutions adopting immersive platforms will need implementation champions - radiologists, educators, and IT leaders - who can advocate for their sustained use and refinement.

## Conclusions

Virtual reality is no longer a speculative innovation in radiology - it is a clinically validated technology with FDA-cleared applications across diagnostic interpretation, procedural planning, medical education, and interdisciplinary communication. As this review has shown, seven VR platforms currently meet regulatory standards for use in radiology: V3D-Explorer, V3D-Vascular, V3D-Colon, VSI HoloMedicine, Ceevra Reveal 3+, Dextroscope, and Dextrobeam. These systems represent a focused subset of the 69 virtual and augmented reality devices cleared by the FDA as of 2024. While the broader list spans many medical domains, this review emphasizes the immersive tools specifically designed to enhance radiologic interpretation, planning, and education. Collectively, these seven platforms enable enhanced spatial reasoning, real-time manipulation of volumetric data, collaborative procedural planning, and immersive teaching environments. Preliminary evidence suggests that V3D-Explorer and V3D-Vascular support improved diagnostic workflows; VSI HoloMedicine and Ceevra Reveal 3+ facilitate surgical decision-making and patient engagement; V3D-Colon enhances colonoscopic training and detection; and Dextroscope and Dextrobeam contribute to neurosurgical simulation and interdisciplinary radiologic learning.

Still, broader clinical integration will require technical refinement, PACS interoperability, robust outcome-driven studies, and incorporation into residency curricula. As virtual reality moves from demonstration to deployment, the shift from flat-screen interpretation to immersive 3D visualization reflects not just a technological advance, but a fundamental evolution in how radiologists understand and communicate imaging data. With sustained innovation, validation, and institutional support, VR is positioned to become a transformative and enduring extension of radiology’s diagnostic, procedural, and pedagogical practice.
